# Prediction of clinical efficacy of acupuncture intervention on upper limb dysfunction after ischemic stroke based on machine learning: a study driven by DSA diagnostic reports data

**DOI:** 10.3389/fneur.2024.1441886

**Published:** 2025-01-07

**Authors:** Yaning Liu, Yuqi Tang, Zechen Li, Pei Yu, Jing Yuan, Lichuan Zeng, Can Wang, Su Li, Ling Zhao

**Affiliations:** ^1^School of Acu-Mox and Tuina, Chengdu University of Traditional Chinese Medicine, Chengdu, China; ^2^School of Automation, Chongqing University, Chongqing, China; ^3^Department of Radiology, Hospital of Chengdu University of Traditional Chinese Medicine, Chengdu, China; ^4^Sichuan Provincial Acupuncture Clinical Medicine Research Center, Chengdu, China; ^5^Key Laboratory of Acupuncture for Senile Disease, Ministry of Education, Chengdu, China

**Keywords:** stroke, machine learning, DSA, AutoGluon, upper limb dysfunction, acupuncture

## Abstract

**Objective:**

To develop a machine learning-based model for predicting the clinical efficacy of acupuncture intervention in patients with upper limb dysfunction following ischemic stroke, and to assess its potential role in guiding clinical practice.

**Methods:**

Data from 1,375 ischemic stroke patients with upper limb dysfunction were collected from two hospitals, including medical records and Digital Subtraction Angiography (DSA) reports. All patients received standardized acupuncture treatment. After screening, 616 datasets were selected for analysis. A prediction model was developed using the AutoGluon framework, with three outcome measures as endpoints: the National Institutes of Health Stroke Scale (NIHSS), Fugl-Meyer Assessment for Upper Extremity (FMA-UE), and the Modified Barthel Index (MBI).

**Results:**

The prediction model demonstrated high accuracy for the three endpoints, with prediction accuracies of 84.3% for NIHSS, 77.8% for FMA-UE, and 88.1% for MBI. Feature importance analysis identified the M1 segment of the Middle Cerebral Artery (MCA), the origin of the Internal Carotid Artery (ICA), and the C1 segment of the ICA as the most critical factors influencing the model’s predictions. Notably, the MBI emerged as the most sensitive outcome measure for evaluating patient response to acupuncture treatment. Additionally, secondary analysis revealed that the number of sites with cerebral vascular stenosis (specifically 1 and 3 sites) had a significant impact on the final model’s predictions.

**Conclusion:**

This study highlights the M1 segment, the origin of the ICA, and the C1 segment as key stenotic sites affecting acupuncture treatment efficacy in stroke patients with upper limb dysfunction. The MBI was found to be the most responsive outcome measure for evaluating treatment sensitivity in this cohort.

## Introduction

Stroke is a sudden loss of neurological function caused by a disturbance in cerebral blood circulation, and it is recognized by the World Health Organization as the second leading cause of death globally (1). Previous studies have indicated that the global burden of stroke is increasing annually (2). Stroke is characterized by a high incidence, disability rate, mortality rate, recurrence rate, and economic burden, contributing to a rising number of elderly individuals with disabilities (3). Residual limb dysfunction, particularly upper limb motor impairment, is a common sequela in the later stages of stroke recovery, severely affecting daily activities and significantly reducing quality of life (4). Clinical research has shown that recovery of lower limb function occurs much more rapidly than that of the upper limbs in stroke patients (5). After active treatment, 80% of patients still experience varying degrees of fine motor dysfunction in the upper limbs (6). Furthermore, 37% of patients continue to face challenges with upper limb motor control 3 months after the stroke, with only 5–20% achieving near-normal hand function (7). Effectively promoting recovery of upper limb dysfunction after stroke remains a significant challenge.

Currently, rehabilitation is the primary treatment for limb motor dysfunction after ischemic stroke. Acupuncture, as a safe, effective, and cost-efficient traditional rehabilitation therapy, is widely used for treating motor, sensory, speech, cognitive, and other dysfunctions post-stroke (8, 9). It has shown significant efficacy in promoting limb function recovery (10), improving abnormal muscle tone, and facilitating fine motor recovery of the hand. Compared to modern medical treatments for motor dysfunction after stroke, acupuncture has the advantages of personalized diagnosis and treatment, rapid response, and diverse therapeutic strategies (9). Additionally, acupuncture demonstrates superior outcomes for managing pain, muscle spasms, sensory dysfunction, and other comorbid symptoms (11).

In recent years, data analysis technologies and artificial intelligence have provided new directions for clinical research. Machine learning methods have found broad applications in fields such as imaging diagnosis, disease prognosis, and big data healthcare (12). The individualization of acupuncture treatment adds complexity to related data-driven research, making traditional analytical methods insufficient for comprehensively exploring acupuncture’s clinical efficacy. Machine learning, as a flexible tool for handling complex medical data (13), has made strides in acupuncture research. For instance, Huo et al. (14) used deep learning techniques to analyze acupuncture treatment for neck pain and cervical spondylosis, while Yin et al. (15) explored machine learning approaches to predict acupuncture efficacy in treating Functional Dyspepsia (FD), paving the way for optimizing personalized acupuncture treatment plans. These studies have successfully integrated clinical diagnosis and treatment data with machine learning technology to predict acupuncture’s therapeutic outcomes and disease prognosis. However, the application of machine learning in acupuncture research is still limited, and there are no studies focused on stroke. Thus, it is crucial to further investigate the feasibility of using machine learning to analyze acupuncture clinical data in stroke patients.

To adapt to the complexity of medical data, prediction models and hyperparameters have become increasingly diversified. The challenge of fine-tuning prediction models has led to the concept of AutoML in artificial intelligence (16). The stacking model, a prominent method in AutoML, typically outperforms single-model training (17). What sets AutoML apart from traditional stacking models is its ability to optimize hyperparameters for each model involved (18).

Imaging diagnosis of stroke has reached a high level of maturity, with common methods including CT, MRI, and DSA (19, 20). As the gold standard for diagnosing cerebrovascular disease (21), DSA plays a crucial role in the diagnosis of ischemic stroke. With the widespread adoption of DSA, nearly all ischemic stroke patients who undergo standardized treatment are diagnosed using this method. While CT and MRI are primarily used to determine the location and extent of lesions, DSA offers clear visualization of stenosis, occlusion, and collateral circulation of cerebral vessels (22). DSA enables rapid and accurate diagnosis of cerebrovascular diseases, helping physicians assess disease progression, formulate treatment plans, and analyze prognosis. Therefore, a thorough analysis of DSA diagnostic data is essential for evaluating treatment methods related to stroke. In this study, we combined clinical efficacy evaluation data with DSA diagnostic reports and employed machine learning to develop a prediction model for acupuncture efficacy, aiming to explore its potential to guide clinical practice.

## Data and methods

### Sample data

The sample data were obtained from the Hospital of Chengdu University of Traditional Chinese Medicine and the Fifth People’s Hospital of Chengdu. This research has been approved by the Medical Ethics Committee of the Hospital of Chengdu University of Traditional Chinese Medicine (Ethics Approval No. 2023KL-023).

### Inclusion and exclusion criteria

The diagnostic criteria were based on the International Classification of Diseases (ICD-11) and the 2018 Chinese Guidelines for the Diagnosis and Treatment of Acute Ischemic Stroke (23). The inclusion criteria were as follows: (1) A confirmed diagnosis of ischemic stroke, based on imaging (MRI or CT), with an onset within 1–14 days; (2) First-time unilateral hemispheric ischemic stroke; (3) Age between 18 and 80 years, regardless of gender; (4) No history of head surgery, thrombolysis, or thrombectomy treatment following stroke onset; (5) Presence of upper limb dysfunction, classified as Brunnstrom stage II–V; (6) The patient received standardized acupuncture treatment during the acute phase, with a treatment duration exceeding 14 days; (7) The patient underwent Digital Subtraction Angiography (DSA) within 7 days of hospital admission.

Exclusion criteria were as follows: (1) Incomplete medical record data that could not meet the requirements of the study; (2) Presence of other serious organic diseases that could contribute to limb dysfunction, such as multiple sclerosis, traumatic brain injury, or spinal cord injury.

The standard treatment was based on secondary stroke prevention (24) and basic nursing care, employing the Xingnao Kaiqiao acupuncture protocol (25). Secondary stroke prevention included respiratory support, anti-platelet aggregation, improvement of cerebral blood circulation, neuroprotective therapy, control of risk factors, infection prevention, and treatment of complications. The primary acupuncture points for Xingnao Kaiqiao therapy were PC6 (Neiguan), DU26 (Shuigou), SP6 (Sanyinjiao), DU20 (Baihui), GB20 (Fengchi), LU5 (Chize), HT1 (Jiquan), and LI4 (Hegu). Additional acupoints could be added or removed based on the individual patient’s condition, but adjustments were made according to the syndrome differentiation related to upper limb dysfunction. Therefore, it was necessary for trained professional practitioners to screen the data during the subsequent data processing phase. During hospitalization, patients received daily acupuncture sessions, each lasting 30 min, with a total of over 10 treatments. The acupuncture was administered by an experienced practitioner with more than 5 years of expertise.

### Establishment of sample data

The sample data were collected from medical records and diagnostic reports of inpatients in the Neurology Departments of two hospitals. A total of 1,375 ischemic stroke patients treated between 2020 and 2023 were included. The data consists of three components: basic demographic information (gender, age), DSA diagnostic reports, and efficacy evaluation metrics.

The standardized DSA diagnostic report includes image characteristics, procedural details, postoperative treatments, and diagnostic conclusions. The primary data for this study includes the location and stenosis ratio of cerebral artery stenosis as indicated in the diagnostic report. The anatomical sites were classified and coded according to DSA cerebrovascular segmentation, which includes: (1) Aortic arch classification; (2) C1-C7 segments of the left and right Internal Carotid Artery (ICA), and the ICA bifurcation; (3) M1-M4 segments of the left and right Middle Cerebral Artery (MCA); (4) A1-A5 segments of the left and right Anterior Cerebral Artery (ACA); (5) V1-V5 segments of the left and right Vertebral Artery (VA), and the VA origin; (6) P1-P4 segments of the left and right Posterior Cerebral Artery (PCA); (7) Left and right Common Carotid Artery (CCA); (8) Left and right Subclavian Artery (SCA); (9) Basilar artery.

The primary efficacy evaluation indices in this study were the Fugl-Meyer Assessment for Upper Extremity (FMA-UE), Modified Barthel Index (MBI), and National Institutes of Health Stroke Scale (NIHSS). FMA-UE is a well-established scale for assessing upper limb movement disorders post-stroke (26). MBI reflects improvements in upper limb function through the evaluation of patients’ daily living abilities (27), while NIHSS provides a comprehensive assessment of stroke severity (28, 29). Efficacy data were collected from the original medical records at admission and discharge.

Three specialists were selected to review the original data of 1,375 stroke patients, following strict inclusion and exclusion criteria. Ultimately, 616 cases met the criteria and were included for further analysis. To protect patient privacy, sensitive information, including patient details and DSA diagnostic data, was anonymized. The review process was divided equally between two doctors, with cross-validation conducted after all assessments to minimize subjectivity and errors. In the case of disagreements, a third specialist intervened for final adjudication. After data correction, the dataset was established and imported into Python for subsequent analysis.

**Figure 1:**
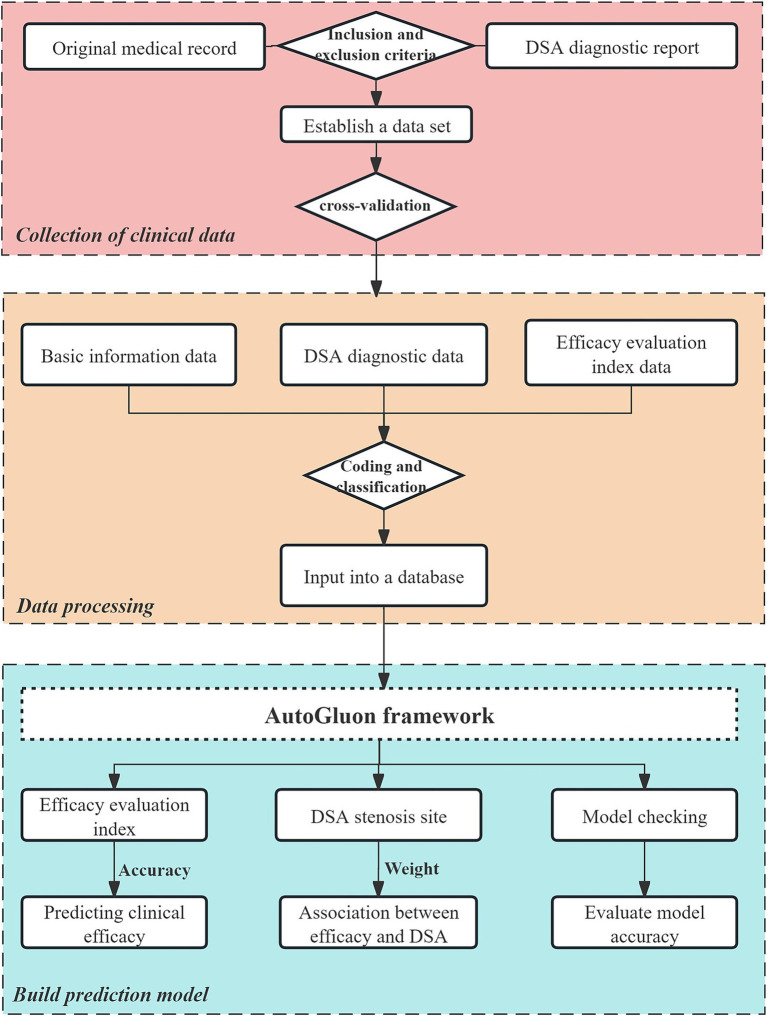
Flowchart of the data processing designed in this paper.

This study developed prediction models using the AutoGluon framework. The model incorporated primary demographic data (age, gender), DSA diagnostic data (stenosis location and ratio), and admission data for the three efficacy evaluation indices (NIHSS, FMA-UE, MBI). The prediction endpoint was the discharge evaluation data for these same indices. The data processing flow is illustrated in Figure 1.

### Data processing and machine learning

#### Data preprocessing

Data preprocessing involves several steps, including data alignment, handling missing values, and converting data formats. Medical record and DSA diagnostic report data are extracted in strict accordance with the predefined coding schema.

#### The establishment of prediction model

**Figure 2:**
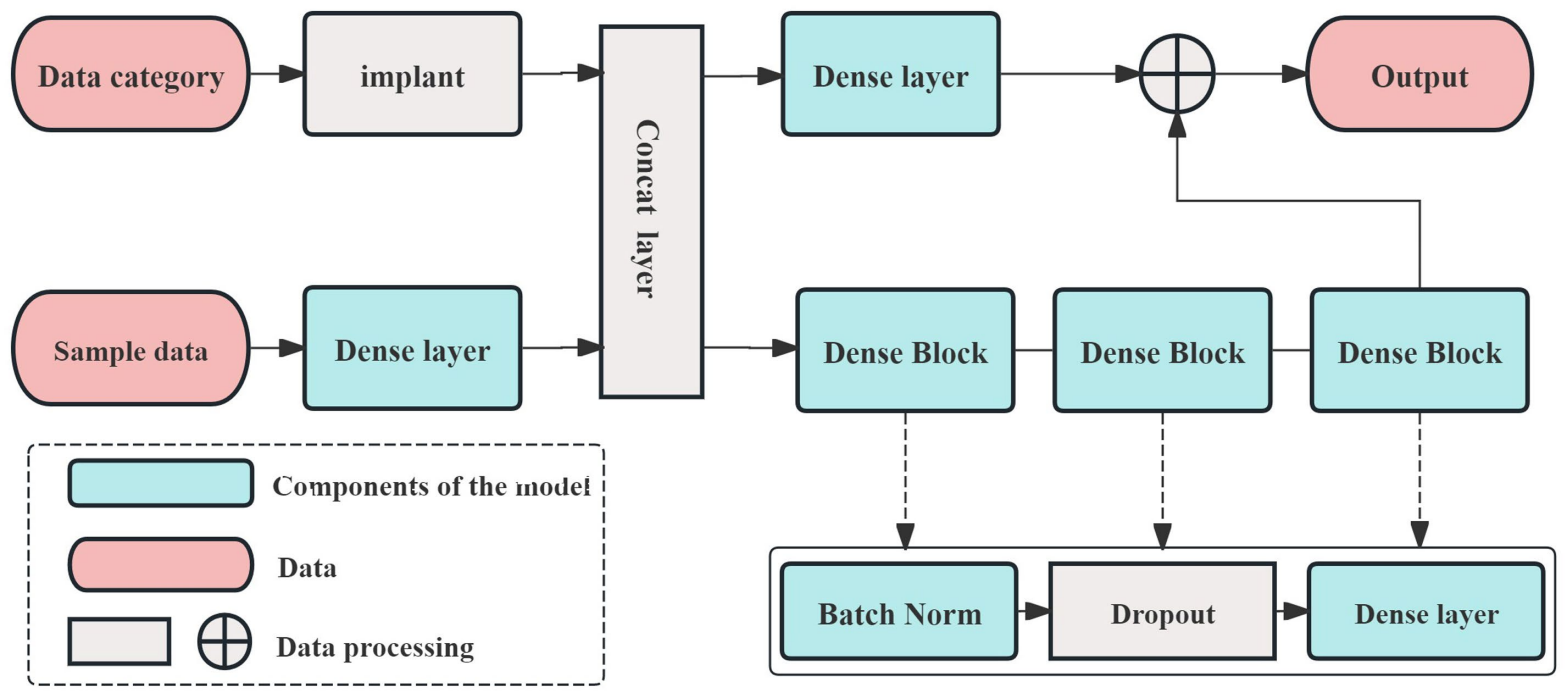
The network structure of the AutoGluon framework.

The prediction model in this study is built using the AutoGluon framework (30, 31). AutoGluon utilizes an optimized k-fold cross-validation method, which helps prevent overfitting, making it particularly suitable for small sample datasets. In this study, 70% of the samples were randomly assigned to the training set, with the remaining 30% used for testing. Additionally, AutoGluon operates modularly, allowing for the easy integration of necessary models during the training process. The framework also facilitates out-of-fold predictions by stacking models, which enhances the accuracy and interpretability of the prediction results. The network architecture designed for this study, based on the AutoGluon framework, is shown in Figure 2.

In Figure 2, the dense layer performs three key functions. The first function involves the calculation of weights, which can be described as follows:

(1) y=Wx

In Equation 1, *x* represents the input data, *y* denotes the output data, and *W* is the learnable weight of the model, which is updated through backpropagation.

Any inevitable bias during training is described as follows:

(2) y=Wx+b

In Equation 2, *b* represents the bias term. The final step involves determining the activation function, which enables the model to function properly and is expressed in Equation 3:

(3) $$\mathrm{out}=\mathrm{activate}\left(y\right)$$

**Figure 3:**
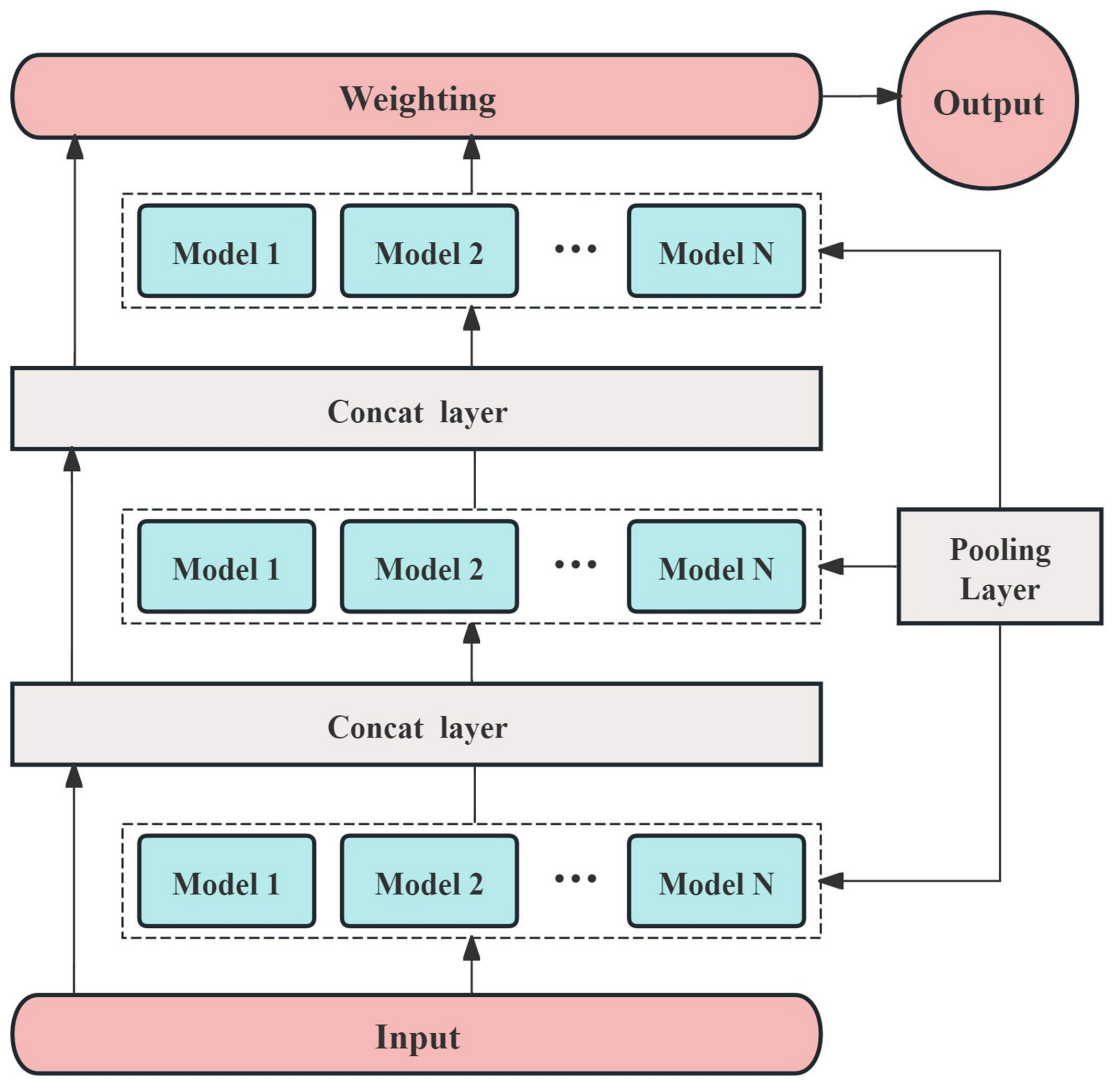
The experimental framework of prediction model.

Different activation functions may perform better for specific tasks or models, but most are designed to suit a limited range of models. Given that the AutoGluon framework supports a variety of prediction models, we selected ReLU as the activation function for this study. To accommodate the small sample size, we constructed a multi-model, three-layer AutoGluon network for the experiment, as shown in Figure 3. In addition to the default models in AutoGluon, including random forests, extremely randomized trees, and k-nearest neighbors, the pooling layer incorporates 12 additional prediction models: linear regression, logistic regression, polynomial regression, ridge regression, support vector machines (with polynomial, linear, and Gaussian kernel functions), decision trees, and AdaBoost.

## Results

### Basic information

The basic information includes patient ID, name, clinic visit time, age, and gender. Patient ID, name, and clinic visit time do not impact the diagnosis or treatment process. Given the limited categories for age and gender, we performed frequency analysis for data processing. The average age of the patients is 65, with a male-to-female ratio of 2:1. The outcome measures did not follow a normal distribution, therefore, the Wilcoxon rank-sum test was used for statistical analysis. The detailed results are presented in Table 1.

**Table 1:** Statistical analysis of outcome indicators.

	Pre-treatment median	Post-treatment median	Pre-treatment interquartile range	Post-treatment interquartile range	*p*-value
FMA	3	2	5	4	<0.001
NIHSS	50	29	58	16	<0.001
MBI	79	86	46	28	<0.001

### The prediction accuracy of the model

**Figure 4:**
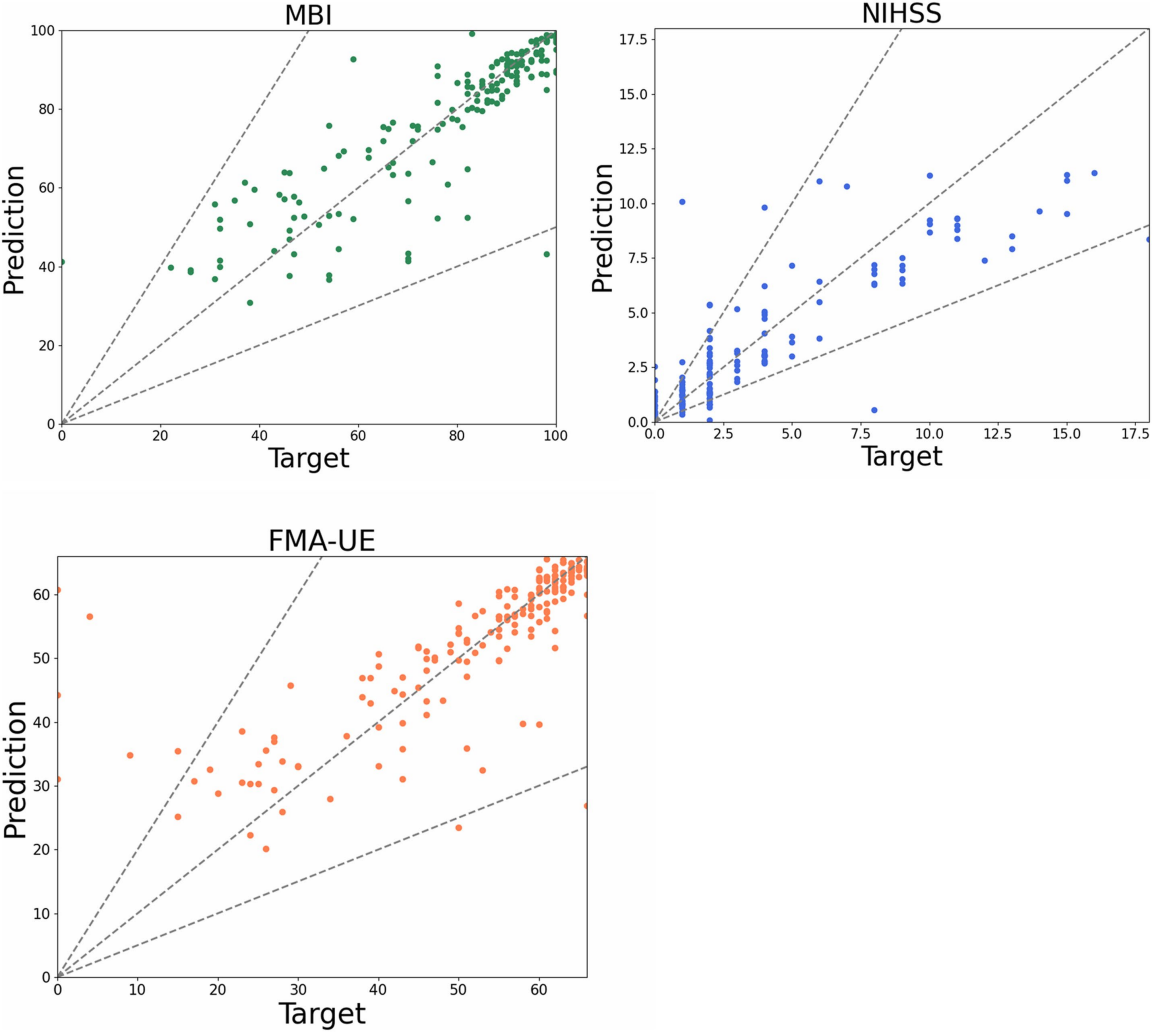
Scatter plot of prediction model. The dots in the figure represent the predicted value of each patient, the funnel area formed by the dotted line represents the acceptable error interval, and the closer the dotted line to the diagonal represents the more accurate the predicted value.

Based on the medical record data of 616 qualified patients, the prediction model was developed using the AutoGluon framework. The model incorporated primary information (age, gender), DSA diagnosis report data (stenosis location, stenosis ratio), and admission evaluation data from three efficacy indices (NIHSS, FMA-UE, MBI). The prediction endpoints were the discharge evaluation data for these same indices. Model parameters were set according to clinical evaluation experience and the characteristics of the efficacy indices. When NIHSS was used as the endpoint, the error value was 1.14, with a prediction accuracy of 84%. For FMA-UE, the error value was 5.15, and accuracy was 78%. When MBI was the endpoint, the error value was 6.34, with accuracy reaching 88% (Figure 4).

### Weight of cerebrovascular stenosis

Building upon the three evaluation index systems from the previous studies, we developed three distinct prediction models and extracted the weights for each eigenvalue corresponding to the cerebrovascular stenosis sites. Given the large number of eigenvalues associated with cerebrovascular stenosis locations, only those with higher weights are presented for clarity.

When FMA-UE is used as the predicted clinical efficacy evaluation index, the corresponding eigenvalue weights are presented in Table 1. The results indicate that the right M1 segment, the origin of the left ICA, and the left M1 segment are the three most influential eigenvalues in the prediction model. For the MBI and NIHSS prediction models, the eigenvalue weights are also shown in Table 2, with the right M1 segment, the left C1 segment, and the origin of the right ICA being the key factors driving the model’s predictions. Across the three models based on different clinical efficacy evaluation indices, the right M1 segment consistently holds the highest weight. When considering the impact of bilateral cerebrovascular locations, the M1 segment, the origin of the ICA, and the C1 segment emerge as the most critical eigenvalues affecting all three prediction models.

**Table 2:** Eigenvalues obtained by using prediction model.

Features	Classification	Weights		
		NIHSS	FMA-UE	MBI
Aortic arch classification	/	8.027393	30.664173	39.434749
The beginning of the ICA	Left	5.380544	30.96917	34.187723
	Right	6.217616	22.654181	37.382045
C1	Left	6.251932	22.160501	37.904647
	Right	5.020586	14.511791	27.64551
C2	Right	2.731205	/	/
C4	Left	/	8.446753	10.292958
C5	Left	2.047457	/	/
C6	Right	2.352296	9.300368	15.404309
C7	Left	2.304183	10.21609	14.800577
	Right	2.16399	12.807273	13.993051
M1	Left	5.406385	23.719628	32.219586
	Right	7.352477	32.14579	55.975485
M2	Left	4.815944	13.600412	14.236988
	Right	2.420018	8.375285	15.923479
A1	Left	/	8.736165	11.08119
The beginning of the VA	Left	2.968495	11.171888	21.107765
	Right	5.485185	17.20229	26.422733
Base artery	/	2.453823	10.367592	19.15955

As shown in Table 1, the weight of the ICA (C1-C7) segment decreases with increasing segment number. Although the weights of the base artery and vertebral artery (VA) are relatively low, they still exert varying degrees of influence on all three prediction models. Additionally, the aortic arch classification, which serves as a defining indicator for each dataset, is assigned a higher weight in all three models.

**Figure 5:**
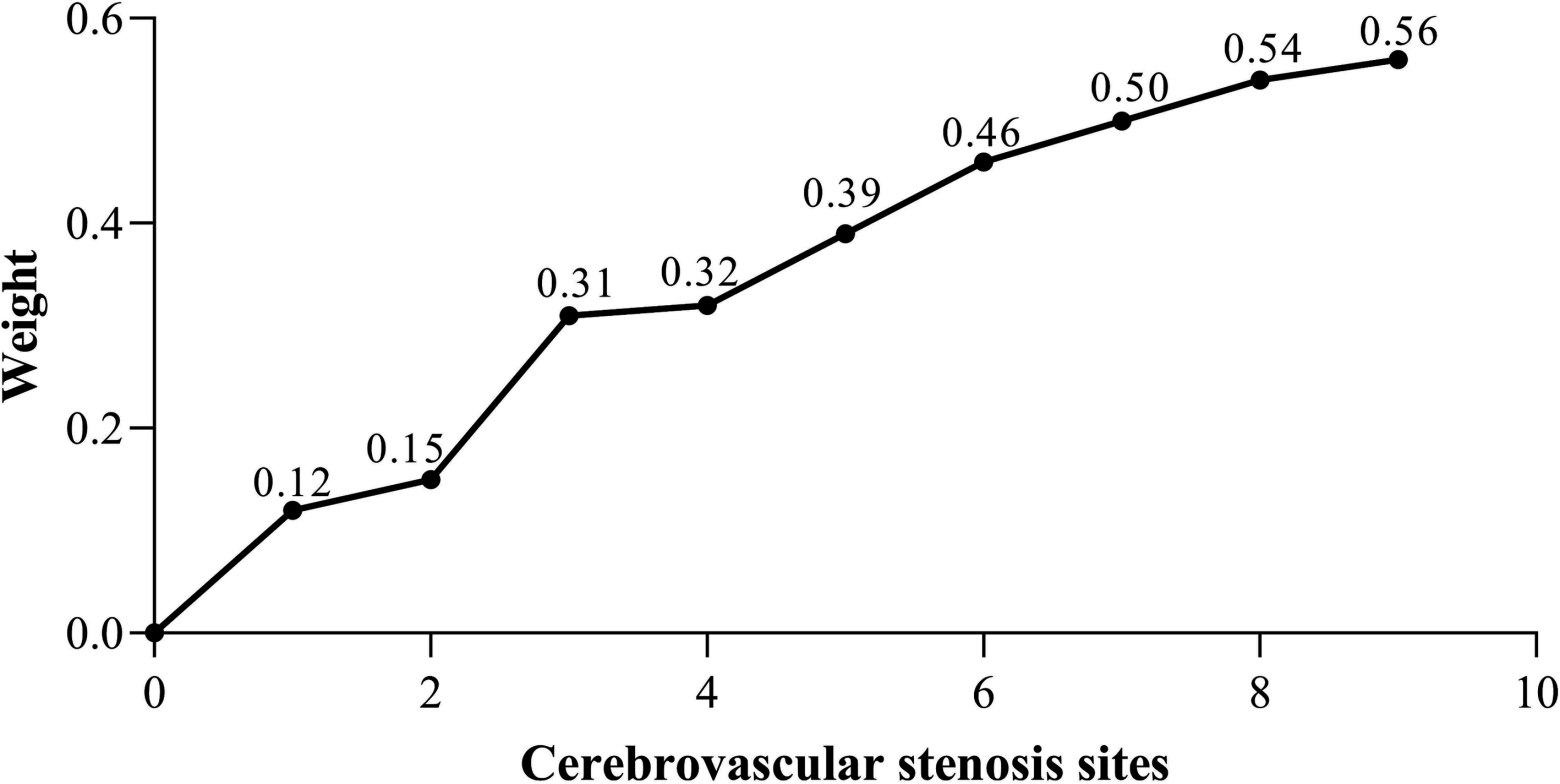
Eigenvalues weights of 9 sub data sets.

The sample data for this study were collected from clinically measured patient data, with cerebrovascular stenosis information obtained from clinical DSA diagnostic reports. As such, the data is highly complex and high-dimensional, particularly with respect to the location and severity of cerebrovascular stenosis, exhibiting significant heterogeneity among patients. To analyze the relationship between the location of cerebrovascular stenosis and acupuncture efficacy, we divided the dataset based on the number of stenosis sites in each patient and performed a secondary analysis. The statistical results indicated that patients had between one and nine stenosis sites. Consequently, the dataset was split into nine sub-datasets, each corresponding to a specific number of stenosis sites, with each sub-dataset treated as an independent feature set. To maintain the integrity of the experiment, the data categories and specific data within the sub-datasets remained consistent with the original dataset. Additionally, the same prediction model used in the previous experiment was applied to ensure consistency in the data analysis approach. Finally, the prediction model generated eigenvalue weights for each of the nine sub-datasets, as shown in Figure 5.

The results indicate that the most significant increase in weight occurred when the number of cerebrovascular stenosis sites increased from 0 to 1 and from 2 to 3. This suggests that having one or three stenosis sites is a critical factor influencing weight change. When the number of stenosis sites exceeds three, the weight increase becomes more gradual, with the effect of additional stenosis sites on the weight diminishing. Furthermore, the weight difference between three and four stenosis sites was found to be the smallest.

### Weight of efficacy evaluation index

**Figure 6:**
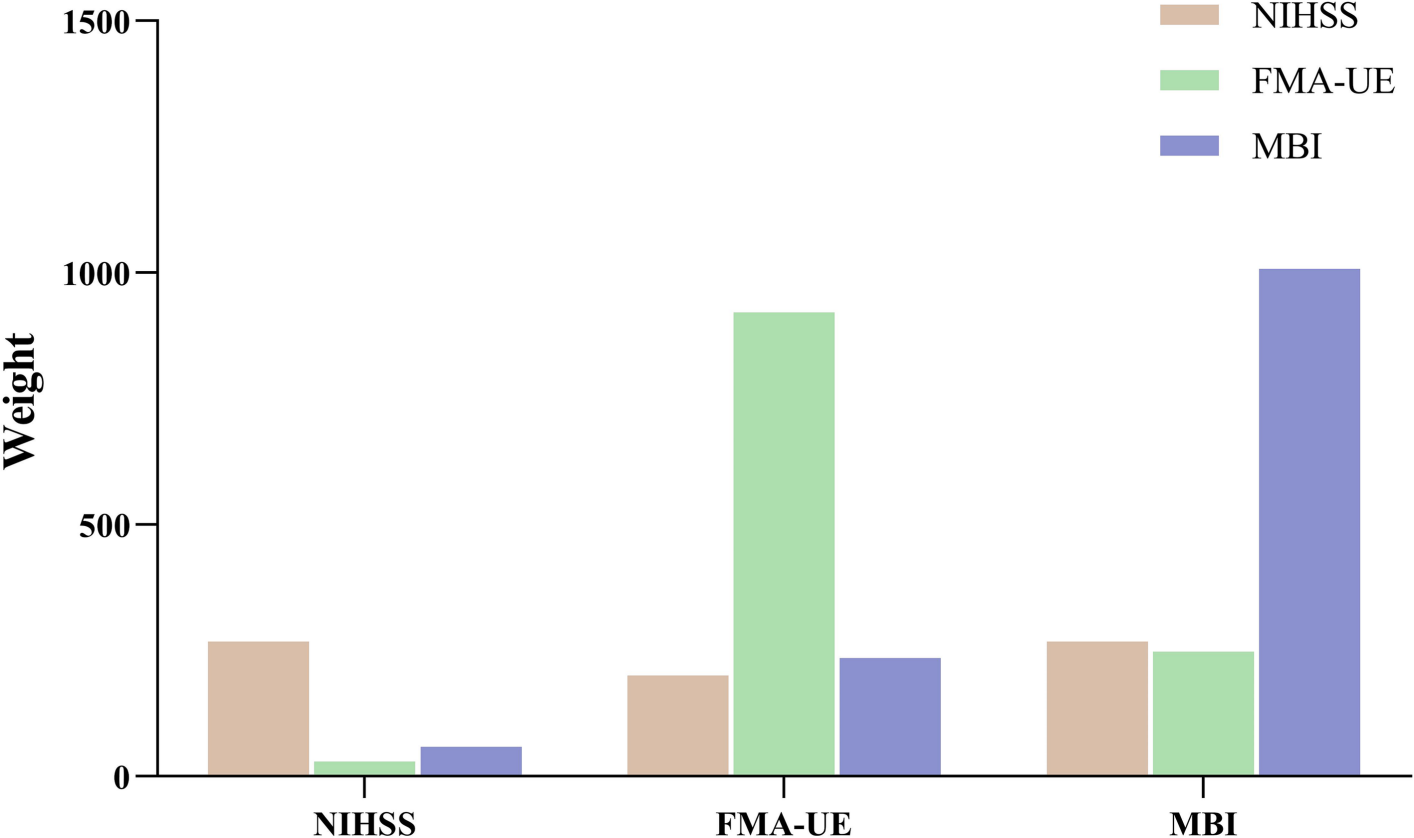
Eigenvalues weights of Efficacy evaluation index.

To assess the feasibility of the efficacy evaluation indices in the prediction model, the admission data based on these indices were incorporated as one of the features in the model. The resulting weight values for each evaluation index are presented in Figure 6. The findings reveal that, when using each evaluation index for prediction, its weight is the highest, without significant influence from the other two indices. These results confirm that the predictions of the three efficacy evaluation indices align with clinical expectations, with MBI exhibiting the highest weight. Specifically, when NIHSS is used as the predictor, MBI’s weight exceeds that of FMA-UE; when FMA-UE is used as the predictor, MBI’s weight surpasses that of NIHSS; and when MBI is the predictor, NIHSS’s weight is higher than that of FMA-UE. Additionally, when comparing the weights of the three predictor groups horizontally, NIHSS shows the lowest overall weight, while FMA-UE and MBI demonstrate relatively high and closely aligned weight distributions.

### Nomogram prediction of acupuncture response

**Figure 7:**
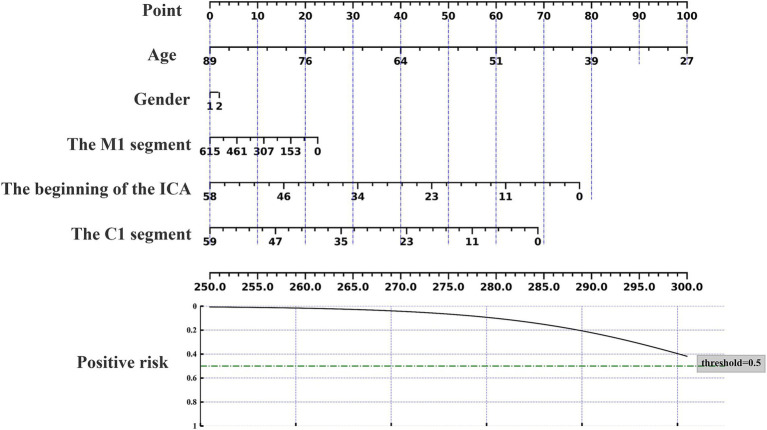
Nomogram for predicting the location characteristics of cerebrovascular stenosis based on FMA-UE.

**Figure 8:**
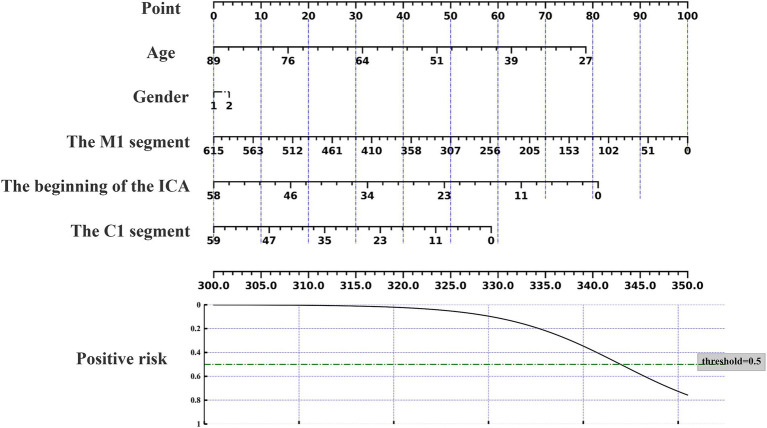
Nomogram for predicting the location characteristics of cerebrovascular stenosis based on MBI.

**Figure 9:**
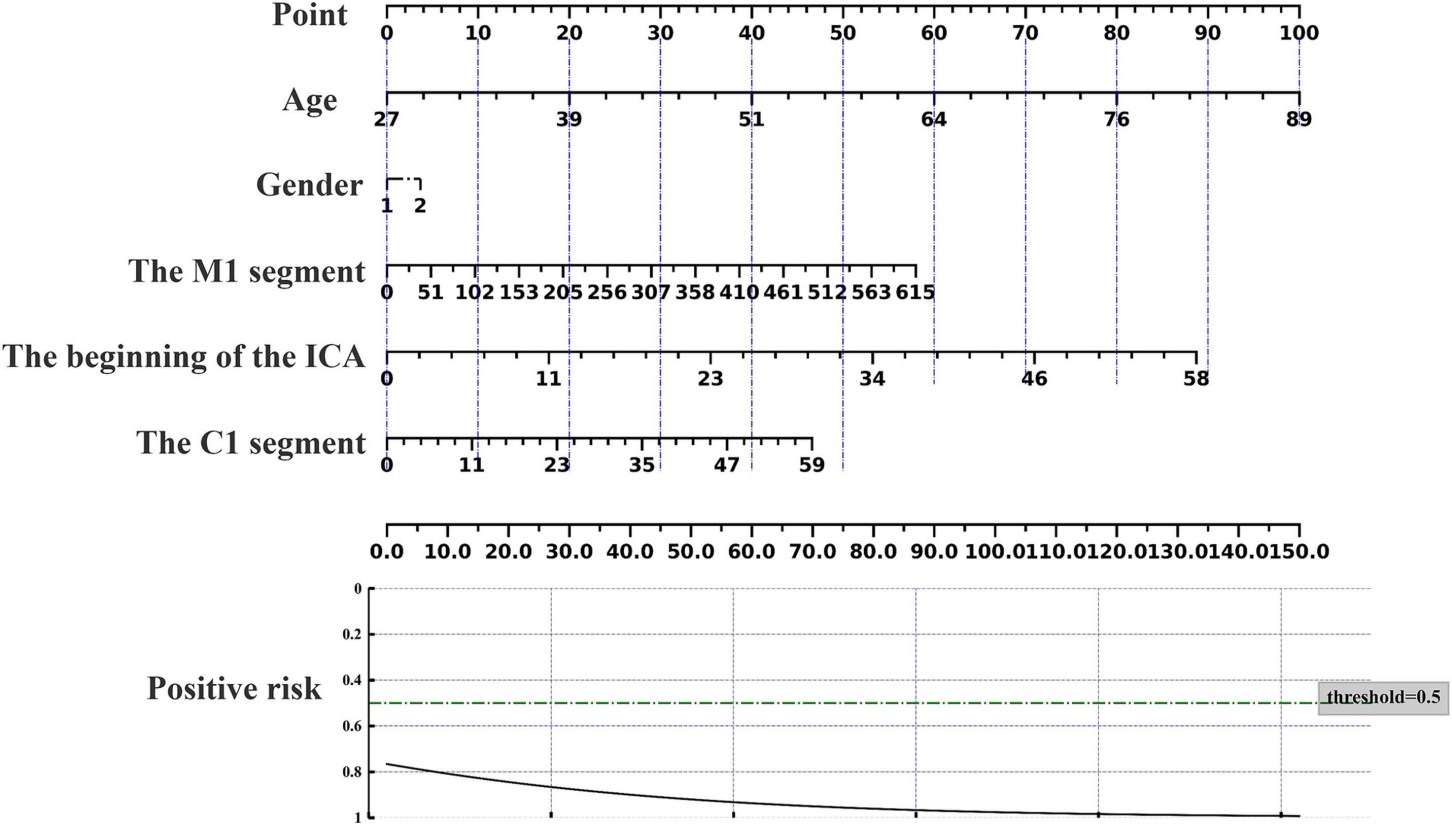
Nomogram for predicting the location characteristics of cerebrovascular stenosis based on NIHSS.

The nomogram can be used to predict the probability of response to acupuncture treatment for upper extremity motor dysfunction following a stroke (Figures 7–9). The total score is obtained by summing the points assigned to each variable, and the response probability is predicted based on the total score. Age is categorized as “1” for females and “2” for males. The values of three key cerebrovascular stenosis locations are represented by a scale based on their corresponding weight values. We constructed three nomograms based on three outcome measures—FMA-UE, NIHSS, and MBI—focusing on upper extremity motor function, neurological deficits, and activities of daily living, to guide clinical decision-making.

## Discussion

This study successfully leveraged the AutoGluon framework and 33 cerebrovascular features derived from DSA diagnostic reports to predict improvements in clinical symptoms and quality of life in stroke patients with upper limb dysfunction, both at the start and end of treatment. Key stenosis sites, including the M1 segment, the origin of the ICA, and the C1 segment, were identified as significant factors influencing the effectiveness of acupuncture treatment for upper limb dysfunction following ischemic stroke. Additionally, the MBI emerged as the most sensitive measure for evaluating these patients. These findings provide valuable insights into the clinical application of acupuncture for stroke-related limb dysfunction. Furthermore, the predictive model developed in this study offers personalized acupuncture treatment recommendations, which can optimize therapeutic outcomes and help alleviate the economic burden of early-stage diagnosis for stroke patients.

This study focuses on patients with upper limb dysfunction after stroke treated with acupuncture. In addition to patient selection, the choice of acupuncture protocol is crucial. Unlike randomized controlled trials, which ensure uniform interventions, clinicians often use individualized treatment plans. To maintain authenticity, we based the protocol on the commonly used Xingnao Kaiqiao method for acute ischemic stroke, which has been proven safe and effective (32), with its standardized approach widely recognized internationally (33).

Due to the limited sample size and high-dimensional data, we adopted the stacking model (AutoGluon framework) to build the prediction model. Traditional algorithms in medical research, such as Random Forest (34), Support Vector Machine (35), and KNN (36), are often used with smaller datasets and offer advantages for clinical studies with limited samples. However, these algorithms rely on manually selected features, which can significantly impact their generalization performance, making them less suitable for high-dimensional data. In contrast, the AutoGluon framework stacks 12 prediction models and automatically selects the most suitable ones based on the data characteristics, enhancing the reliability of the analysis. This approach, widely applied in machine learning, has seen increasing use in the medical field in recent years. For example, Byeon et al. (37) developed a multi-omics model for COVID-19 severity prediction using AutoGluon, and Bo et al. (38) used it to predict responses to Lenvatinib Monotherapy for unresectable hepatocellular carcinoma. This study is the first to apply AutoGluon in acupuncture-related data analysis, yielding promising results.

**Figure 10:**
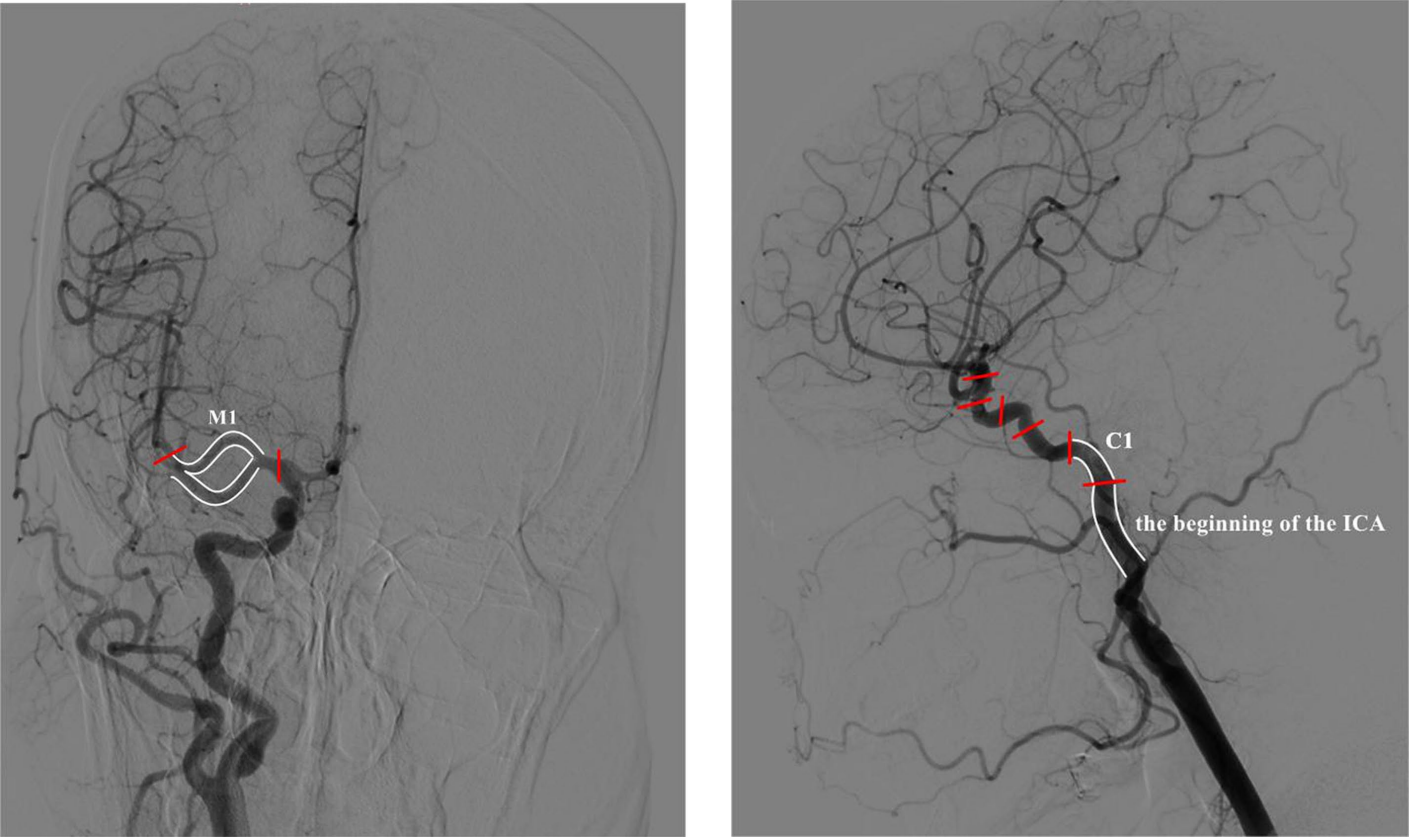
Normal DSA schematic diagram.

We developed prediction models using three efficacy evaluation indices (NIHSS, FMA-UE, MBI) to predict the impact of acupuncture on upper limb motor dysfunction after ischemic stroke, achieving high prediction accuracy, with MBI reaching 88%. The model analysis identified three cerebrovascular stenosis sites that significantly influenced the predictions: the M1 segment, the origin of the ICA, and the C1 segment, with the M1 segment having the highest weight (Figure 10). These sites are common stenosis locations in ischemic stroke patients and correspond to areas of the brain responsible for limb motor function, aligning with clinical practice. This suggests that acupuncture may offer better therapeutic outcomes for patients with stenosis in these regions.

Further analysis of cerebrovascular stenosis revealed that the number of stenosis sites most significantly impacted the model when there were 1, 3, or 4 sites, as shown in Figure 5. These findings suggest that patients with fewer than four stenosis sites, including one or more of the M1, ICA origin, or C1 segments, may be more suitable candidates for acupuncture treatment. Additionally, sensitivity analysis of the clinical efficacy indices showed that MBI had the highest sensitivity, followed by FMA-UE and NIHSS. MBI has been shown to be a reliable tool for evaluating acute stroke patients (27), with high sensitivity in detecting even minor improvements in daily living abilities (39, 40). These results demonstrate the potential of DSA diagnosis for screening patients, assisting clinicians in tailoring individualized acupuncture treatments, improving patient outcomes, and minimizing unnecessary use of medical resources.

This study introduces new clinical data to explore, for the first time, the relationship between acupuncture’s effect on upper limb dysfunction after ischemic stroke and cerebrovascular conditions. To optimize data analysis, we employed the flexible stacking model (AutoGluon framework), which enhances the reliability of our results. However, several limitations remain. First, further evidence is needed to support the association between acupuncture efficacy and cerebrovascular stenosis location, including the use of Arterial Spin Labeling (ASL) for evaluating cerebral vascular remodeling (41) and Diffusion Tensor Imaging (DTI) for assessing cerebral structural plasticity (42). Additionally, our study focused on stenosis of larger vessels and did not evaluate microcirculation or collateral circulation. Future studies will address this gap through DSA image analysis. Furthermore, due to the small sample size, the strength of the evidence is limited, and the reliability of the sample size test model should be improved in subsequent research. Future randomized controlled trials based on our study’s inclusion and exclusion criteria will help verify whether these findings are applicable in clinical practice.

## Conclusion

The M1 segment, the origin of the ICA, and the C1 segment are key stenosis sites that significantly influence the effectiveness of acupuncture treatment in patients with upper limb motor dysfunction following ischemic stroke. Additionally, the MBI demonstrated the highest sensitivity in evaluating these patients.

## Data Availability

The original contributions presented in the study are included in the article/Supplementary material, further inquiries can be directed to the corresponding author.
